# Inhibition of renalase expression and signaling has antitumor activity in pancreatic cancer

**DOI:** 10.1038/srep22996

**Published:** 2016-03-14

**Authors:** Xiaojia Guo, Lindsay Hollander, Douglas MacPherson, Ling Wang, Heino Velazquez, John Chang, Robert Safirstein, Charles Cha, Fred Gorelick, Gary V. Desir

**Affiliations:** 1 Department of Medicine, VACHS, Yale University School of Medicine, New Haven, CT 06520, USA; 2 Renal Division, Renji hospital, Shanghai Jiaotong Univ School of Medicine, Shanghai, China; 3 Department of Surgery, VACHS, Yale University, New Haven, CT 06520, USA; 4 Department of Surgery, University of Connecticut, Farmington, CT 06032, USA

## Abstract

An essential feature of cancer is dysregulation of cell senescence and death. Renalase, a recently discovered secreted flavoprotein, provides cytoprotection against ischemic and toxic cellular injury by signaling through the PI3K-AKT and MAPK pathways. Here we show that renalase expression is increased in pancreatic cancer tissue and that it functions as a growth factor. In a cohort of patients with pancreatic ductal adenocarcinoma, overall survival was inversely correlated with renalase expression in the tumor mass, suggesting a pathogenic role for renalase. Inhibition of renalase signaling using siRNA or inhibitory anti-renalase antibodies decreased the viability of cultured pancreatic ductal adenocarcinoma cells. In two xenograft mouse models, either the renalase monoclonal antibody m28-RNLS or shRNA knockdown of renalase inhibited pancreatic ductal adenocarcinoma growth. Inhibition of renalase caused tumor cell apoptosis and cell cycle arrest. These results reveal a previously unrecognized role for the renalase in cancer: its expression may serve as a prognostic maker and its inhibition may provide an attractive therapeutic target in pancreatic cancer.

Pancreatic cancer is one of the most lethal neoplasms, and causes approximately 330,000 annual deaths globally, with 40,000 in the US^1^. Pancreas cancer is difficult to detect, and most cases are diagnosed at a late stage^2^. Although there has been some progress in the use of chemotherapy of this cancer, the disease remains extremely resistant to all drugs therapies^3^. The overall 5 year survival for individuals with pancreatic cancer is <5%^3^, and additional therapeutic targets are needed.

The development of pancreatic cancer relies on the stepwise accumulation of gene mutations^4^, some of which cause abnormal MAPK, PI3K and JAK-STAT signaling. Progression from minimally dysplastic epithelium to dysplasia to invasive carcinoma reflects the stepwise accumulation of gene mutations that either activate oncogenes (e.g. *KRAS2*), or inactivate tumor suppressor genes (e.g. *CDKN2a/INK4a*, *TP53* and *DPC4/SMAD*4)^5^. Ninety-five, 90 and 75% of pancreatic tumors carry mutations in *KRAS2, CDKN2a*, and *TP53*, respectively. These mutations result in sustained and dysregulated proliferation that characterizes cancer growth. The mutational landscape and core signaling pathways in pancreatic ductal adenocarcinoma (PDAC) have been defined through a comprehensive genetic analysis of 24 advanced PDACs^4^. These data indicate that most PDACs contain a large number of genetic changes that are primarily point mutations, which affect approximately 12 cell signaling pathways.

That study also identified five hundred and forty one genes overexpressed in PDAC by at least 10-fold in 90% of the tumors. This included a 2- to 4-fold increase in the recently characterized protein, renalase (RNLS), in tumors or in tumor derived cell lines. RNLS, a novel secreted flavoprotein^6^,^7^,^8^,^9^ with NADH oxidase activity^10^,^11^, promotes cell and organ survival^12^ through a receptor-mediated process that is independent of its intrinsic enzymatic activities^13^. RNLS rapidly activates protein kinase B (AKT), the extracellular signal-regulated kinase (ERK), and the mitogen activated protein kinase (p38). Chemical inhibition of either ERK or AKT abrogated the protective effect of RNLS^13^. We previously identified the critical region of RNLS that mediates its cytoprotective effects, and showed that a 20 amino acid RNLS peptide (RP-220, aa 220–239: CIRFVSIDNKKRNIESSEIG) is conserved in all known isoforms, but is devoid of any detectable oxidase activity. This peptide was equally effective as recombinant RNLS (rRNLS) at conferring protection against toxic and ischemic injury in the human kidney cell line HK-2 and in mice^13^.

Using biotin transfer studies with RP-220 in the human proximal tubular cell line HK-2 and protein identification by mass spectrometry, we identified PMCA4b as a renalase binding protein. This previously characterized plasma membrane ATPase is involved in cell signaling and cardiac hypertrophy. Co-immunoprecipitation and co-immunolocalization confirmed protein-protein interaction between endogenous renalase and PMCA4b. Down-regulation of endogenous *PMCA4b* expression by siRNA transfection or inhibition of its enzymatic activity by the specific peptide inhibitor caloxin1b each abrogated RP-220 dependent MAPK signaling and cytoprotection. In control studies, these maneuvers had no effect on epidermal growth factor mediated signaling, confirming specificity of the interaction between PMCA4b and renalase. These data indicate that PMCA4b functions as a renalase receptor, and a key mediator of renalase dependent MAPK signaling^14^.

Since RNLS functions as a survival factor that engages the MAPK and PI3K pathways that are disordered in pancreatic cancer, and because its expression is regulated by the signal transducer and activator of transcription STAT3^15^, we postulated that abnormal regulation of RNLS expression and signaling could provide a survival advantage to cancer cells, and promote tumor formation^16^.

Here we provide evidence for both a pathogenic role of increased RNLS expression in PDAC and the potential therapeutic utility of inhibiting RNLS signaling. In addition, we explore the molecular mechanisms that mediate the observed antitumor activity of inhibitors of RNLS signaling.

## Results

### RNLS overexpression in PDAC and association with decreased survival

**Figure 1:**
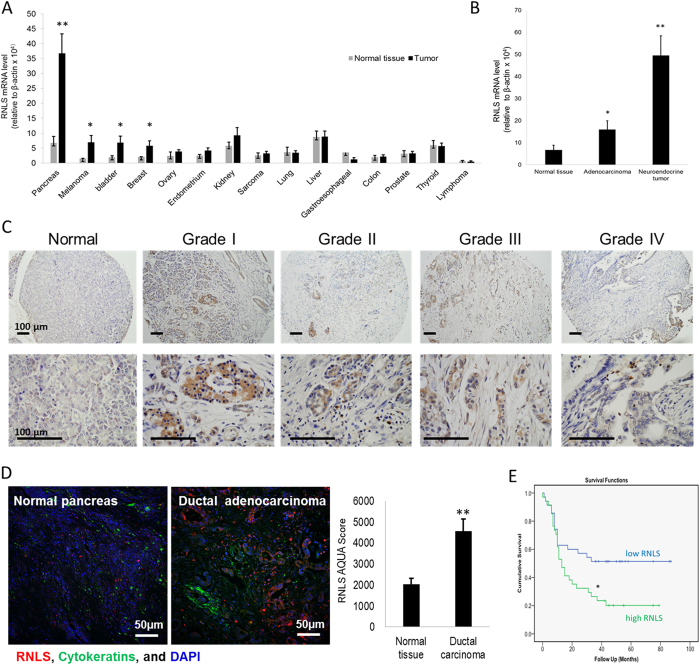
RNLS overexpression in cancer, and association with poor patient outcome in PDAC. (**A**) RNLS mRNA level measured by qPCR in cDNA arrays containing 182 human tumor samples (OriGene Technologies) from 15 different tumor types; *indicates p < 0.05, **indicates p = 0.0001. (**B**) RNLS mRNA level measured by qPCR in normal pancreas (n = 6), pancreatic ductal adenocarcinomas (n = 11), and pancreatic neuroendocrine tumors (n = 23); *indicates p = 0.05; **indicates p = 0.00017. (**C**) RNLS protein expression detected by immunohistochemistry using m28-RNLS in normal human pancreatic tissue (*left panel*, n = 90), ductal adenocarcinoma (Grades 1–4, n = 20 each); representative result shown for each; RNLS protein stains brown. (**D**) RNLS expression detected using anti-RNLS-m28 for immunofluorescence staining of tissue microarray of normal human pancreatic tissue (*left panel*, n = 90), ductal carcinoma (*middle panel*, n = 90); representative result shown for each, and blue color: nuclei, green color: cytokeratin, and red color: RNLS; *right panel*: fluorescence intensity quantified using the AQUAnalysis^TM^ software, normal human pancreatic tissue (n = 90), ductal carcinoma (n = 90), ***indicates p = 0.00013. (**E**) Kaplan-Meier survival curve for survival rates; Biomax cohort of 69 PDACs stratified into low (n = 35, RNLS AQUA score < median) and high (n = 34, RNLS AQUA score > median) RNLS expression, *indicates p = 0.0001.

To determine if *RNLS* expression differed between normal and cancer tissue, we examined fifteen different types of cancer by screening commercially available human tissue cDNA arrays using quantitative PCR (qPCR). *RNLS* expression was significantly increased in cancers of the pancreas, bladder, and breast and in melanoma (Fig. 1A). Because of their particularly poor survival and limited therapeutic options, we focused our attention on pancreatic neoplasms. RNLS expression was elevated in both PDAC (~3 fold) and neuroendocrine (8 fold) tumors (Fig. 1B). Immunocytochemical studies using the anti-RNLS monoclonal m28-RNLS showed that RNLS expression was present in PDAC grade 1–4 and was predominantly localized to cancer cells, as shown in Fig. 1C and Supplement Figure 1S. Most RNLS appeared to have a cytoplasmic distribution in cancer cells; it was present in all tumor grades, but was most evident in more-differentiated cancers (Grades I–III). In neuroendocrine tumors of the pancreas, RNLS was expressed in cells throughout the tumor (Supplement Fig. 2S). *RNLS* gene expression was increased in pancreatic ductal adenocarcinoma cell (PDAC) lines with *KRAS2* mutations (MiaPaCa2 and Panc1) compared to those with wild type *KRAS2*, such as BxPC3 (Supplement Fig. 3S).

We characterized RNLS expression in 69 patients with PDAC, using tissue microarrays (TMA) consisting of formalin-fixed, paraffin-embedded tumor cores with matched adjacent normal tissue. The demographics and clinical characteristics of the individuals from whom the samples were obtained are shown in Supplement Table 1S. Examination of 138 histospots from paired PDAC tumors and their non-tumor adjacent tissues for RNLS protein expression, using an unbiased, quantitative, automated immunofluorescence microscopy system (AQUA)^17^, showed that overall RNLS levels were more than 2-fold greater in PDAC tumors than in their adjacent non-tumor pancreatic tissue (p < 0.001, Fig. 1D).

To determine whether enhanced RNLS expression might affect PDAC’s clinical behavior, we asked if its level of expression affected prognosis. Individuals whose tumors expressed high RNLS levels (n = 34 with RNLS AQUA score > median) had a dramatically reduced 3-year survival rate (24% versus 49%, p = 0.024, Fig. 1E). These findings indicate that tumor levels of RNLS expression may be useful prognostic markers in PDAC, and help identify a subset of patients with a more aggressive phenotype.

### Inhibitors of RNLS signaling block pancreatic cancer growth

**Figure 2:**
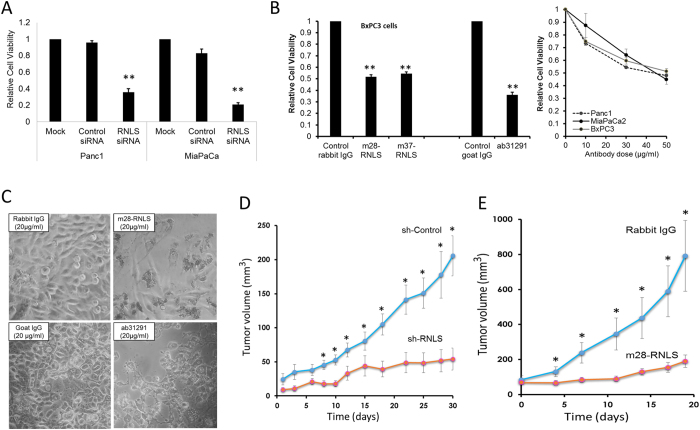
Inhibition of RNLS signaling is cytotoxic to cancer cells *in vitro* and *in vivo*. (**A**) Transient transfection of Panc1 cells using a RNLS-specific siRNA, or a non-specific control siRNA, and cell viability assayed 96 hrs later using the WST-1 reagent; n = 6, **indicates p < 0.001. (**B**) Cells were treated with indicated antibodies for 72 hrs and cell viability determined using WST-1; m28-RNLS and m37-RNLS: monoclonal antibodies raised against RNLS peptide RP220, ab31291: Abcam polyclonal antibody raised against a partial sequence of RP-220; n = 6, *indicates p < 0.005. (**C**) Representative photos of MiaPaCa2 cells after 3 days incubation with m28-RNLS, n = 10. (**D**) Athymic nude mice received subcutaneous injection of Panc1 cells transduced with RNLS shRNA (sh-RNLS) or control (sh-Control); tumor volume measured every 23 days for up to 30 days, n = 6 each; *indicates p < 0.05. (**E**) Nude mice xenografted with BxPC3, tumor volume measured prior to treatment every 3–4 days with 2 mg/kg of either rabbit IgG as a negative control or with m28-RNLS, n = 14, *indicates p < 0.05.

To determine the functional consequences of inhibiting *RNLS* expression and signaling in pancreatic cancer cells, the effect of decreasing RNLS expression on cell viability *in vitro* was evaluated by *RNLS* knockdown using siRNA. This treatment markedly reduced the viability of the PDAC lines Panc1 and MiaPaCa2 (Fig. 2A and Supplement Fig. 4S). Since the RNLS peptide RP-220 mimics the protective effect and signaling properties of rRNLS, we reasoned that it likely interacts with a critical region of the receptor for extracellular RNLS, and that antibodies generated against it could be inhibitory. From a panel of monoclonal antibodies in rabbit against RP-220, two clones, m28-RNLS, and m37-RNLS, were selected based on their high binding affinity (KD of 0.316 and 2.67 nM respectively). The inhibitory effects of m28-RNLS, m37-RNLS, and of a commercially available polyclonal (against a partial sequence of RP-220) on PDAC growth are shown by the representative examples depicted in Fig. 2B,C.

To determine if inhibition of RNLS signaling affected tumor growth *in vivo*, we used shRNA to generate two stably transfected Panc1 cell lines: one containing non-targeting shRNA (sh-Control), and another with RNLS-targeting shRNA (sh-RNLS). *RNLS* expression in sh-RNLS cells was decreased by ~80%, as assessed by qPCR (Supplement Figure 5S). Interestingly, cell cycle regulator *p21* level was significantly increased in the RNLS knockdown stable line (Supplement Figure 5S). The transfected cells were injected subcutaneously into athymic nude mice, and tumor size was assessed over a 30-day period. The tumor volume generated by sh-RNLS cells was significantly smaller than that of sh-Control cells from day 8 until day 30 when the animals were sacrificed (Fig. 2D). Using qPCR, we confirmed that *RNLS* expression remained inhibited in the sh-RNLS tumor xenografts at the time of sacrifice (71.1 ± 5.3% of sh-control, n = 6, p = 0.021).

To evaluate the therapeutic potential of inhibitory antibodies, BxPC3 cells were subcutaneously injected into athymic nude mice, which were treated with either control rabbit IgG or m28-RNLS, and tumor volume was measured for up to 3 weeks. As shown in Fig. 2E, compared to rabbit IgG, m28-RNLS treatment caused a significant decrease in tumor volume.

### Induction of apoptosis and cell cycle arrest in tumor cells by m28-RNLS

**Figure 3:**
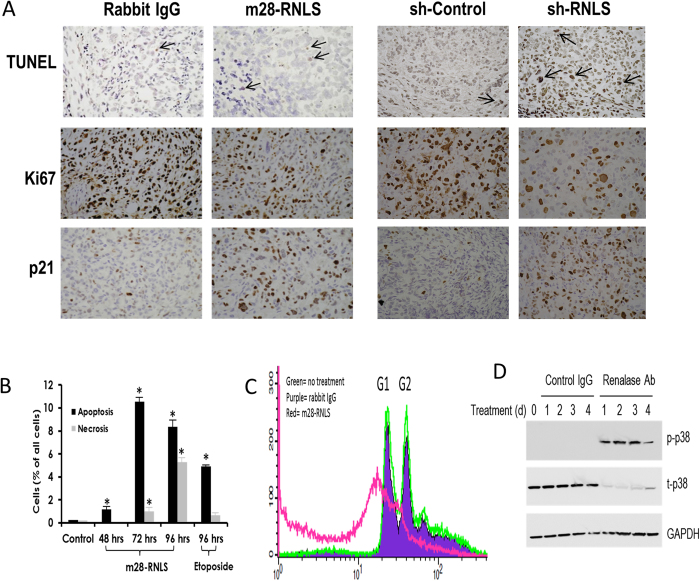
Inhibition of RNLS signaling induces apoptosis and cell cycle arrest. (**A**) Representative images of sections from BxPC3 xenografted tumors (n = 14 each) treated with anti-m28-RNLS or control rabbit IgG, and of xenografts of Panc1 cells transduced with RNLS shRNA (sh-RNLS) or control (sh-Control). Tissues are stained for TUNEL (arrows: positive cells), cell proliferation marker Ki67 (brownish stain), and cell cycle inhibitor p21 (brownish stain). (**B**) FACS analysis of Panc1 cells in culture treated with either m28-RNLS (30 μg/ml) or 100 μM etoposide (positive control) for 4 days; n = 3, *indicates p < 0.05. (**C**) Effect of m28-RNLS on cell cycle of Panc1 cells determined by FACS analysis; green curve: no treatment, purple curve: rabbit IgG, red curve: m28-RNLS 30 μg/ml. **(D**) Panc1 cells treated with polyclonal ab31291 or with goat IgG as a negative control, and cell lysates probed for activation of p38 by western blot from day 0 to 4. Representative blot, n = 4.

Sections of BxPC3 xenografted tumors from mice treated with either rabbit IgG or m28-RNLS revealed a ~2-fold increase in apoptosis (TUNEL staining) (Fig. 3A, top left panels) in the antibody-treated tumors: m28-RNLS vs IgG; 28.4 ± 3.3 positive cells/high power field vs. IgG- 14.8 ± 2.3, n = 14, *p* = 0.002. A similar increase in TUNEL staining (2.5 fold) was seen in sections of sh-RNLS Panc1 xenografts (Fig. 3A, top right panels). Moreover, m28-RNLS treatment of BxPC3 tumors led to a 2.5-fold decrease in the expression of a cellular proliferation marker Ki67 (m28-RNLS vs IgG: IgG, 137.1 ± 14.9 vs 340.2 ± 11.9 positive cells/high power field, n = 14, p = 1.4 × 10^−8^) (Fig. 3, middle left panels), and to a ~4-fold increase in the expression of the cell cycle regulator p21 expression (m28-RNLS vs IgG: IgG, 178.1 ± 11.4 vs 42.2 ± 4.7.6 positive cells/high power field, n = 14, p = 1.6 × 10^−10^) (Fig. 3A, bottom left panels). Similar changes in Ki67 (2.5 fold decrease) and p21 (4 fold increase) expression were documented in sections of sh-RNLS Panc1 xenografts (Fig. 3A, middle and bottom right panels).

FACS analysis of Panc1 cells was performed to examine the effect of RNLS signaling inhibition on the cell cycle. As shown in Fig. 3B and Supplement Figure 6S, Panc1 cells in culture treated with m28-RNLS undergo apoptosis. The data shown in Fig. 3C confirm that RNLS inhibition caused apoptosis, as evidenced by the appearance of a large pre-G1 peak. They also reveal a marked decrease in G2, indicating that inhibition of RNLS signaling by m28-RNLS causes a pre-G2 cell cycle arrest. The development of apoptosis was temporally associated with p38 phosphorylation (Fig. 3D).

### Presence of a positive RNLS-STAT3 feedback loop and its interruption by m28-RNLS

**Figure 4:**
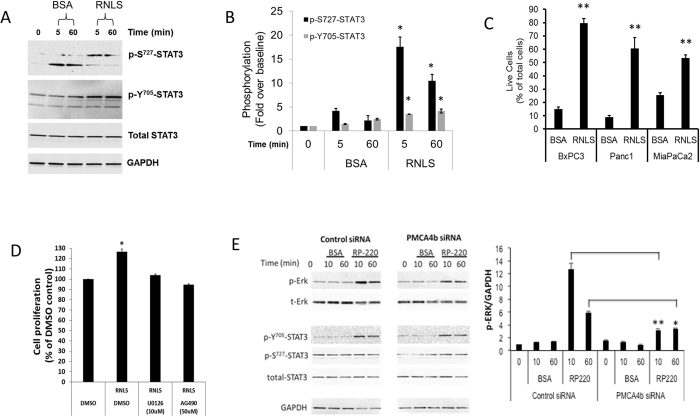
RNLS activates STAT3, and RNLS overexpression favors cancer cell survival. (**A**) Activation of STAT3 by RNLS in Panc1 cells; Panc1 cells in culture treated with either BSA or RNLS, and STAT3 phosphorylation assessed by western blot; p-Ser^727^-STAT3: phosphorylation at serine 727, p-Y^705^-STAT3: phosphorylation at tyrosine 705; representative study. (**B**) Quantification of STAT3 phosphorylation with RNLS; signals normalized to total STAT3; n = 3, *indicates *p* < 0.05. (**C**) PDAC lines BxPC3, Panc1 and MiaPaCa2 are serum starved for 48 hrs, then incubated with 30 μg/ml of either bovine serum albumin (BSA) or rRNLS for 3 days; total and live cell number determined using trypan blue and an automated cell counter; n = 4, **indicates p < 0.0001. (**D**) Effect of rRNLS on survival of Panc1 cells in the absence or presence of 10 μM U0126 (Erk inhibitor) or 50 μM AG490, (JAK2 and STAT3) for 72 h. The doses of AG490 and U0126 chosen had been reported to inhibit the phosphorylation of JAK^37^ and ERK1/2^38^ respectively in Panc1 cells. The time point of 72 hrs was chosen to allow the cell time to proliferate. Cell proliferation was measured by the WST-1 method, and depicted as % change of DMSO-treated Panc1 cells. n = 6, *=p < 0.01. (**E**) siRNA mediated inhibition of PMCA4b expression blocks RNLS mediated MAPK signaling; *Left and middle panels*: MiaPaCa2 cells transfected with either non-targeting or PMCA4b siRNA, maintained in serum free medium for 3 days and treated with either 25 μg of BSA or 25 μg of RNLS peptide RP-220 for the indicated time; RP-220 mediated ERK and STAT3 activation assessed by western blot and representative immunoblots are shown; p-ERK = phosphorylated ERK, p-Y^705^-STAT3 = phosphorylated STAT3, p-S727-STAT3 = phosphorylated STAT3, BSA = bovine serum albumin, RP-220 = RNLS peptide agonist; *Right panel*: quantification of phosphorylated ERK (p-ERK), signals normalized to glyceraldehyde 3-phosphate dehydrogenase (GAPDH) loading control; n = 4, *=P < 0.03, **=p < 0.0001.

STAT3 binds to the promoter region of the *RNLS* gene and increases its expression^15^. A positive RNLS-STAT3 feedback loop is suggested by the observation that in HK-2 cells treated with RNLS, STAT3 phosphorylation at serine 727 (p-Ser^727^-STAT3) and tyrosine 705 (p-Y^705^-STAT3) increases 2 and 4 fold respectively, while STAT1 is unaffected (Supplement, Figure 7S). As depicted in Fig. 4A,B, the addition of RNLS to the PDAC line Panc1 caused a rapid increase in phosphorylated STAT3 (p-Ser^727^-STAT3 and p-Y^705^-STAT3). Additional support for a RNLS-STAT3 feedback loop is provided by the finding that inhibition of RNLS signaling in Panc1 by m28-RNLS leads to a long-lasting and sustained decrease in p-Y^705^-STAT3 (Supplement, Figure 8S and 9S).

### RNLS as a survival factor for pancreatic cancer cells

RNLS-mediated signaling protects HK-2 cells exposed to toxic stress from apoptosis^12^,^13^. To explore if RNLS signaling provided a survival advantage to pancreatic ductal adenocarcinoma cells (PDAC) exposed to stress, serum was withdrawn from cultured BxPC3, Panc1, and MiaPaCa2 cells for 48 hours, and either recombinant RNLS (rRNLS) or bovine serum albumin (BSA) was added to the culture medium for an additional 72 hrs; total and live (trypan blue exclusion) cell counts were determined. Compared to BSA, rRNLS increased PDAC survival rate by 2- to 5-fold (Fig. 4C).

We have shown that the cytoprotection afforded by the addition of rRNLS to HK-2 cells exposed to hydrogen peroxide or cisplatin injury was dependent on ERK activation^12^,^13^. The results shown in Fig. 4D suggest that rRNLS also improves PDAC survival in an ERK- and STAT3-dependent manner since pretreating with U0126, an inhibitor of the MAPK kinase MEK1^18^ and AG490, a compound inhibits JAK2, Erk2, and STAT3 activity^19^, abrogated rRNLS’ protective effect.

Evidence regarding PMCA4b’s role in RNLS dependent signaling in pancreatic cancer was obtained by specifically down-regulating PMCA4b expression using siRNA. In control studies, non-targeting siRNAs affected neither PMCA4b gene expression nor RNLS-mediated ERK phosphorylation (Fig. 4E, left panel). In contrast, PMCA4b-targeting siRNAs decreased gene expression by 90 ± 3% (n = 4), and reduced RNLS dependent ERK phosphorylation by ∼70% (Fig. 4E, right panel). PMCA4b inhibition had no discernable effect on RNLS mediated STAT3 phosphorylation suggesting the existence of an additional RNLS receptor(s).

## Discussion

Taken together, our findings indicate that upregulated RNLS-mediated signaling plays a pathogenic role in PDAC. We showed that high RNLS expression is associated with a two-fold increase in overall 3-year mortality, suggesting that RNLS could serve as a prognostic marker for pancreatic cancer. Furthermore, since RNLS is a secreted protein, it should also be evaluated as a biomarker for the primary detection of tumors or as a surrogate marker for treatment response or recurrence.

A primary mechanism of RNLS mediated cytoprotection appears to be its ability to increase the anti-apoptotic factor Bcl2 and to prevent the activation of effector caspases^13^. Downregulation of RNLS expression by siRNA decreases the viability of Panc1 and MiaPaCa2 cells by 60% and 82% respectively. We chose to perform the shRNA studies in Panc1 cells because we encountered significant difficulties in propagating MiaPaCa2 cells transduced with RNLS shRNA. It is, therefore, not surprising that inhibition of RNLS signaling in Panc1 cells and in tumor xenograft is associated with a marked increase in the rate of apoptosis. Ki-67 expression is used to evaluate levels of cell division, and we interpret the finding that m28-RNLS downregulates Ki-67 expression in xenografts of pancreatic cancer as evidence that RNLS inhibition decreases the proliferative rate of tumors.

Many of the key factors that determine cell cycle progression have been identified. They include cyclin dependent kinases (CDK) and two classes of endogenous CKD inhibitors, namely the inhibitor of cyclin dependent kinase 4 (INK4) and the CDK interacting proteins/kinase inhibitor (CIP/KIP) protein families^20^. Our data reveal that the expression of p21, a CKD inhibitor belonging to the CIP/KIP family, is regulated by RNLS signaling. Inhibition of RNLS signaling is associated with a marked increase in p21 expression. Since p21 is a negative regulator of cell cycle that can maintain cells in G0, block G1/S transition, and cause G1 or inter-S phase arrest^20^, its upregulation could account for the decrease in cell proliferation observed in tumors treated with m28-RNLS. In addition, p38 has also been shown to affect cell cycle progression^21^; its activation by anti RNLS treatment could also contribute to cell cycle arrest.

The regulatory promoter elements and transcription factors that regulate *RNLS* gene expression have been recently investigated^15^, and these data point to a key role for STAT3. Our results suggest a feed-forward loop between RNLS and STAT3: signals that upregulate STAT3 increase *RNLS* gene expression, and RNLS, in turn, increases STAT3 activity. Such an interaction between RNLS and STAT3 has important implications regarding the role of RNLS signaling in the pathogenesis of cancer. STAT family proteins, particularly STAT3, are firmly implicated in the induction and maintenance of an inflammatory microenvironment that facilitates malignant transformation and cancer progression^22^. STAT3 signaling is often persistently activated in cancer cells. Such activation not only drives tumor cell proliferation, but also increases the production of a large number of genes that sustain inflammation in the tumor microenvironment. A STAT3 feed-forward loop between cancer cells and non-transformed and stromal cells has been documented in cancer^23^,^24^,^25^. For instance, STAT3 is constitutively activated in multiple myeloma patients. In the IL-6-dependent human myeloma cell line U266, IL-6 signals through Janus kinases to the activate STAT3, which in turn up-regulates anti-apoptotic factors, and promotes the survival of tumor cells^23^. Likewise, STAT3 is constitutively activated in the majority of pancreatic ductal adenocarcinomas, and appears to be required for the initiation and progression of KRAS2-induced pancreatic tumorigenesis^26^.

The STAT3 pathway and RNLS may also have a role in promoting the most common and important environmental factor in PDAC development: cigarette smoking^27^,^28^,^29^. Nicotine, a key constituent of cigarette smoke, has been shown to enhance the rate of proliferation and angiogenesis in cancers^30^,^31^. Nicotine’s action of tumor growth and metastases is believed to be mediated by its interaction with an acetylcholine receptor alpha-7nACHR resulting in JAK-STAT3 and MEK-ERK1-2 downstream signaling cascades^32^. Nicotine increases RNLS promoter activity through the synergistic action of Sp1 and STAT3^15^.

Our findings identify RNLS as a secreted protein that can promote the survival and growth of PDACs, and provide a framework to further investigate the use therapies that inhibit RNLS for the treatment of cancer. Due to the multiple mechanisms for regulating MAPK, PI3K, and JAK-STAT3, and the crosstalk between pathways, cell fate depends on the dynamic balance and integration of multiple signals. Our data indicate that the inhibition of RNLS signaling will tilt the balance toward cancer cell death.

Our results also raise fundamental issues about a more general role of RNLS and its regulation in cancer. For example, how do the physiologically relevant regulators of RNLS expression become dysregulated in cancer? Will inhibition of RNLS signaling have antitumor effects for other cancers, such as breast, or bladder in which RNLS expression is increased? We find that that RNLS expression is inversely correlated with overall survival in patients with metastatic melanoma, and that inhibition of renalase signaling decreases melanoma growth *in vitro* and *in vivo* (submitted for publication). We have tested the effect of RNLS inhibition (siRNA and or m28-RNLS) on cell survival in 3 human melanoma cell lines A375.S2, SkMel28, SkMel5, and noted a decrease in cell viability of ~90%. Additionally, administration of m28-RNLS to nude mice bearing A375.S2 xenografts caused a 70% reduction in tumor volume, and a >90% decreases in STAT3 activation. We believe that the results obtained from 4 different cell lines, and 2 tumor models provide support for the hypothesis that inhibition of RNLS signaling may be cytotoxic to other tumors. Lastly, does RNLS signal through a PMCA4b alone or multiple membrane receptors? We believe the examination of these questions will deepen our understanding of the renalase pathway, and lead to the discovery of additional therapeutic targets for the management of cancer.

## Experimental Procedures

### Reagents

The human ductal pancreatic adenocarcinoma cell lines BxPC-3, Panc1 and MiaPaCa-2 were obtained from the American Type Culture Collection (ATCC) (Manassas, VA, USA) and maintained as recommended. The p38 and STAT3 blockers SB203580 and Stattic were purchased from Abcam (Cambridge, UK). The JNK inhibitor SP600125 and the ERK inhibitor U0126 were obtained from Sigma Aldrich (St. Louis, MO, USA), and Cell Signaling Technologies (Beverly MA, USA), respectively. Recombinant human RNLS (rRNLS) was expressed, purified, concentrated, and dialyzed against PBS as previously described^33^. Rabbit anti-RNLS monoclonal (AB178700), goat polyclonal anti-RNLS (AB31291), goat IgG and rabbit IgG were purchased from Abcam.

### Synthesis of anti-RNLS monoclonal antibodies m28-RNLS and m37-RNLS

RNLS peptide RP-220 was conjugated to KLH and used to immunize 6 rabbits, and lymphocytes from the spleens of selected animals were fused to myeloma cells for hybridoma generation. Hybridoma supernatants were screened against rRNLS and selected hybridomas were cloned and expanded for antibody purification. The monoclonal antibodies were purified from conditioned hybridoma culture supernatant by protein A affinity chromatography.

Two clones, m28-RNLS and m37-RNLS, were selected based on their high binding affinity (KD of 0.316 and 2.67 nM respectively) as determined using a Biacore T100 system. We determined the nucleotide sequence of m28-RNLS by PCR, then synthesized and cloned it into a mammalian expression vector. The m28-RNLS, synthesized by transient expression into 293-F cells, was purified by protein A chromatography.

### Tissue specimens

Human cancer cDNA arrays (Screen cDNA Arrays I and II, pancreatic cancer cDNA array) were obtained from OriGene Technologies (Rockville, MD, USA). The relevant pathology reports are available online: http://www.origene.com/assets/documents/TissueScan. Human pancreas cancer and normal tissue samples obtained from US Biomax (Rockville, MD, USA) were used for immunohistochemistry or immunofluorescence.

### Quantitative PCR (qPCR)

Relative expression levels of various genes were assessed by qPCR. The mRNA level of RNLS, 2′-5′-oligoadenylate synthetase 1 (OAS1), β-actin and 18s rRNA was assessed using the TaqMan Gene Expression real-time PCR assays (Applied Biosystems, Carlsbad, CA, USA). The results were expressed as the threshold cycle (Ct). The relative quantification of the target transcripts normalized to the endogenous control 18s rRNA or β-actin was determined by the comparative Ct method (ΔCt) and the 2-ΔΔCt method was used to analyze the relative changes in gene expression between the tested cell lines according to the manufacturer’s protocol (User Bulletin No. 2, Applied Biosystems).

### Immunohistochemistry and western blot analysis

Immunohistochemistry was performed as described previously^34^. Briefly tumor tissues were formalin-fixed, paraffin-embedded and cut into 5-μm sections on glass slides. The slides were de-paraffinized and hydrated, followed by antigen retrieval in a pressure cooker containing 10mM sodium citrate, pH6 buffer. The sections were blocked in 3% hydrogen peroxide for 30 min and 2.5% normal horse serum in PBS/0.1% Tween20 for 1 hr followed by incubation with primary antibody and isotype control IgG overnight at 4 °C. The following antibodies were used in this study: m28-RNLS at 500 ng/ml, goat polyclonal anti-RNLS at 250 ng/ml (Abcam, AB31291), rabbit monoclonal anti Ki67 (Vector Lab, VP-RM04, 1:100), rabbit monoclonal anti p21, and phspho-Tyr^705^-Stat3 (Cell Signaling Technologies, #2947, 1:100 and #9145, 1:400, respectively). ImmPRESS peroxidase-anti-rabbit IgG (Vector Laboratories, Burlingame, CA, USA) was used to detect primary antibodies. The color was developed using a Vector DAB substrate kit and tissue was counterstained with hematoxylin (Vector Laboratories). Slides were observed and photographed using an Olympus BX41 microscope and camera (Olympus America Inc, Center Valley, PA, USA).

Western blot analysis was carried out as previously described^13^.

### Tissue microarray

Pancreas tissue microarrays were purchased from US. BioMax. Tissue microarray slides were stained as described previously^35^. In brief, specimens were co-stained with m28-RNLS and mouse monoclonal pan-cytokeratin antibodies (1:100, DAKO M3515) at 4 °C overnight. The secondary antibodies Alexa 488-conjugated goat anti-mouse (1:100, Molecular Probes, Eugene, OR) and Envision anti-rabbit (DAKO) were applied for 1 hr at room temperature. The slides were washed with Tris-buffered saline (three times for 5 minutes), and incubated with Cy5-tyramide (Perkin-Elmer Life Science Products, Boston, MA) and activated by horseradish peroxidase. Cy5 was used because its emission peak (red) is outside of the green-orange spectrum of tissue auto-fluorescence. The slides were sealed with coverslips with Prolong Gold anti-fade reagent containing 4′,6-Diamidino-2-phenylindole to facilitate the visualization of nuclei.

### Cell Viability Assays

Cell viability was assessed by trypan blue exclusion, and cells were counted using a BioRad TC10 automated counter. For some studies, cell viability was determined using the WST-1 reagent (Roche Diagnostics, Indianapolis, IN, USA) as previously described^13^.

### Apoptosis and cell Cycle analysis

For cell cycle analysis, cultured cells were dissociated using 10 mM EDTA, fixed with ice-cold 70% ethanol, digested with RNAse A, and stained with propidium iodide. Propidium staining was detected using a BD FACSCalibur flow cytometer (BD Biosciences, San Jose, CA, USA), and analyzed using CellQuest software.

Apoptosis was detected and quantified as previously done^34^. In brief, cells were stained with FITC-labeled Annexin-V and propidium iodide according to the manufacturer’s instructions (Bender MedSystems, Burlingame, CA, USA). At least 20,000 events were collected on a BD FACSCalibur flow cytometer (BD Biosciences, San Jose, CA, USA) and analyzed using CellQuest software.

### RNA interference

Four individual siRNAs and a siRNA SMART pool targeting RNLS were purchased from Dharmacon (Lafayette, CO, USA). Cells were transfected with RNLS siRNA or a universal negative control siRNA (control siRNA, Dharmacon) using DharmaFECT 4 reagent (Dharmacon) as suggested by the manufacturer. Downregulation of PMCA4b expression by siRNA was carried out as previously described^36^.

To generate a stably transfected Panc1 cell line, cells were transduced with lentivirus (Santa Cruz) carrying either RNLS shRNA (sh-RNLS) or control shRNA (sh-Control) according to the manufacturer’s protocol. Cells were transduced twice to increase shRNA copy number and stable clones were established after selection in 80 μg/ml puromycin for 10 days. Knock-down efficiency was determined by qPCR.

### Mouse xenograft tumor model

Female athymic, 18–20 g nude mice (nu/nu) were obtained from Charles River (Willimantic, CT) and housed in microisolator cages, with autoclaved bedding in a specific pathogen-free facility, with a 12-h light/dark cycle. Animals received water and food ad libitum, and were observed for signs of tumor growth, activity, feeding and pain, in accordance with the study protocol approved by the VACHS IACUC.

Xenograft tumors were established by subcutaneous injection of BxPC3 cells (2 × 10^6^ in 100 μl of PBS, pH 7.6). When the tumors reached a volume of 50–100 mm^3^, the mice were divided into a control group (n = 14 treated with rabbit IgG, 40 μg by intraperitoneal injection (IP), and an experimental group (n = 14) that received m28-RNLS (40 μg IP, every 3 days). Tumor size was measured with digital calipers and volume was calculated according to the formula (length × width^2^) × π/2. In another group of animals (n = 6 each) sh-RNLS or sh-Control Panc1 cells (2 × 10^6^ in 100 μl of PBS, pH 7.6) were injected subcutaneously. These animals received no further treatments, and tumor size and volume were measured for up to 30 days.

At the end of the study, the mice were sacrificed and the tumors were excised and immediately snap-frozen in liquid nitrogen and stored at −80 °C. Apoptosis was examined using the TUNEL assay (Roche *in situ* Apoptosis Detection System), according to the manufacturer’s instructions. Sections were examined by light microscopy and the apoptosis index was determined by counting ≥1000 cells in 5 randomly selected high-power fields (×200 magnification).

### Statistical analyses

The Wilcoxon rank test and the Mann-Whitney test were used for paired and unpaired data, respectively. When appropriate, nonparametric repeated-measures ANOVA (Friedman test) was used to evaluate statistical significance. When the Friedman test revealed statistical significance, Dunn’s test was used for pairwise comparisons. A Kaplan-Meier survival analysis was carried out, and a sample size calculation using an analysis of variance for two groups indicates that a per-group sample size of 32 would permit detection an effect size of 0.3 with 85% power. All data are mean ± standard error of the mean (mean ± SEM), and values of *p* < 0.05 were accepted as a statistically significant difference. Statistical analyses of tissue array data were performed using SPSS® software, version 21.0 (SPSS Inc., Chicago, IL, USA).

## Additional Information

**How to cite this article**: Guo, X. *et al.* Inhibition of renalase expression and signaling has antitumor activity in pancreatic cancer. *Sci. Rep.* **6**, 22996; doi: 10.1038/srep22996 (2016).

## Supplementary Material

Supplementary Information
