# Supplementary material for: Inhibition of renalase expression and signaling has antitumor activity in pancreatic cancer
Source: Sci Rep. 2016 Mar 14;6:22996. doi: 10.1038/srep22996 (PMC4789641; doi:10.1038/srep22996)
Supplement: Supplementary Information [file srep22996-s1.pdf]

Title:

Inhibition of renalase expression and signaling has antitumor activity in pancreatic cancer

Authors:

Xiaojia Guo[1], Lindsay Hollander[1,4], Douglas MacPherson[1], Ling Wang[2], Heino Velazquez[1], John Chang[1], Robert Safirstein[1], Charles Cha[3], Fred Gorelick[1], and Gary V. Desir[1]

INVENTORY OF SUPPLEMENTAL INFORMATION

Figure 1S-9S

Table 1

Supplement 1S

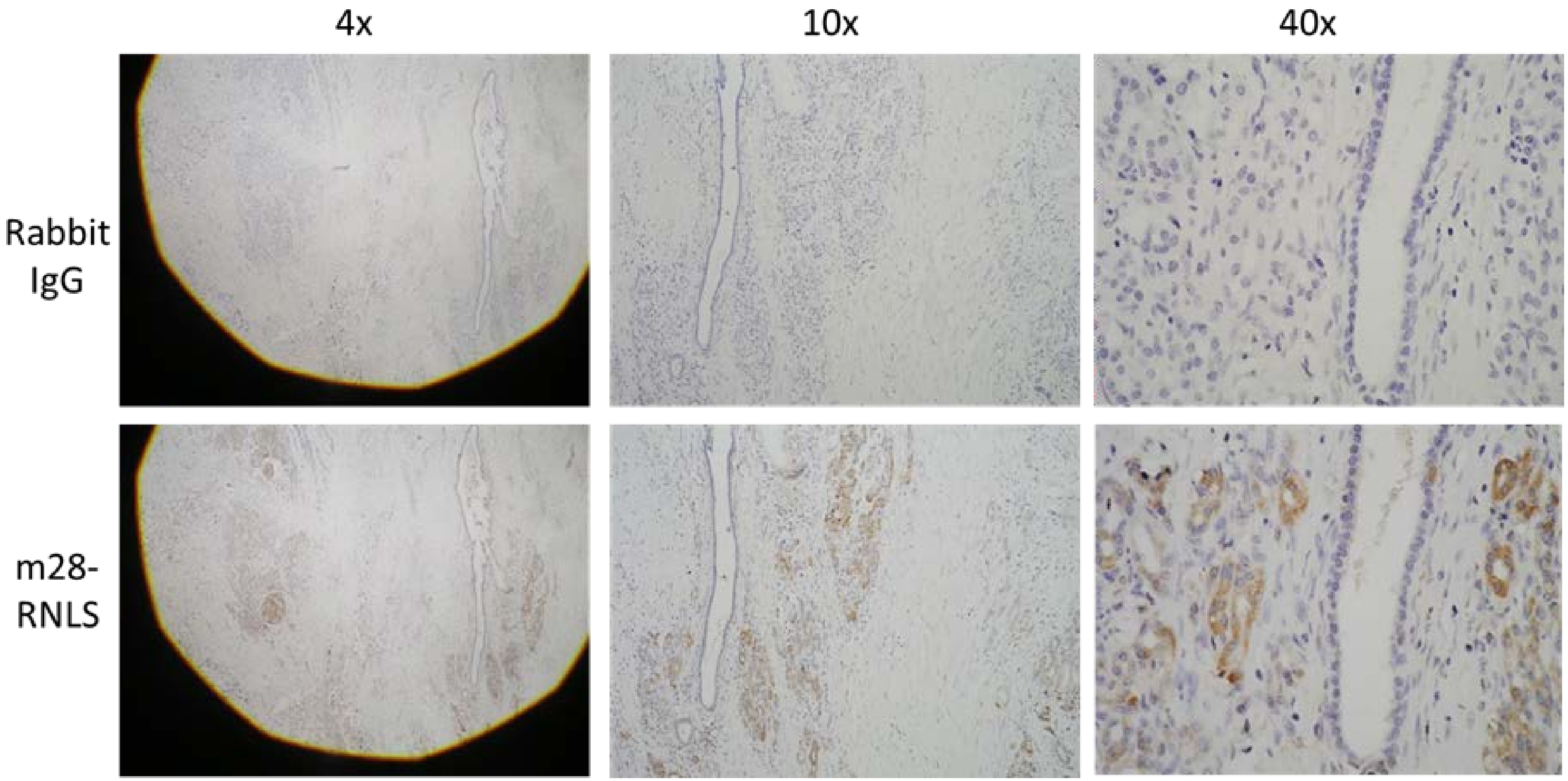

Figure 1S: Localization of RNLS in Grade II PDAC. Representative immunohistochemistry staining for RNLS protein expression (brown) using m28-RNLS in a Grade II human pancreatic ductal adenocarcinoma.

Supplement 2S

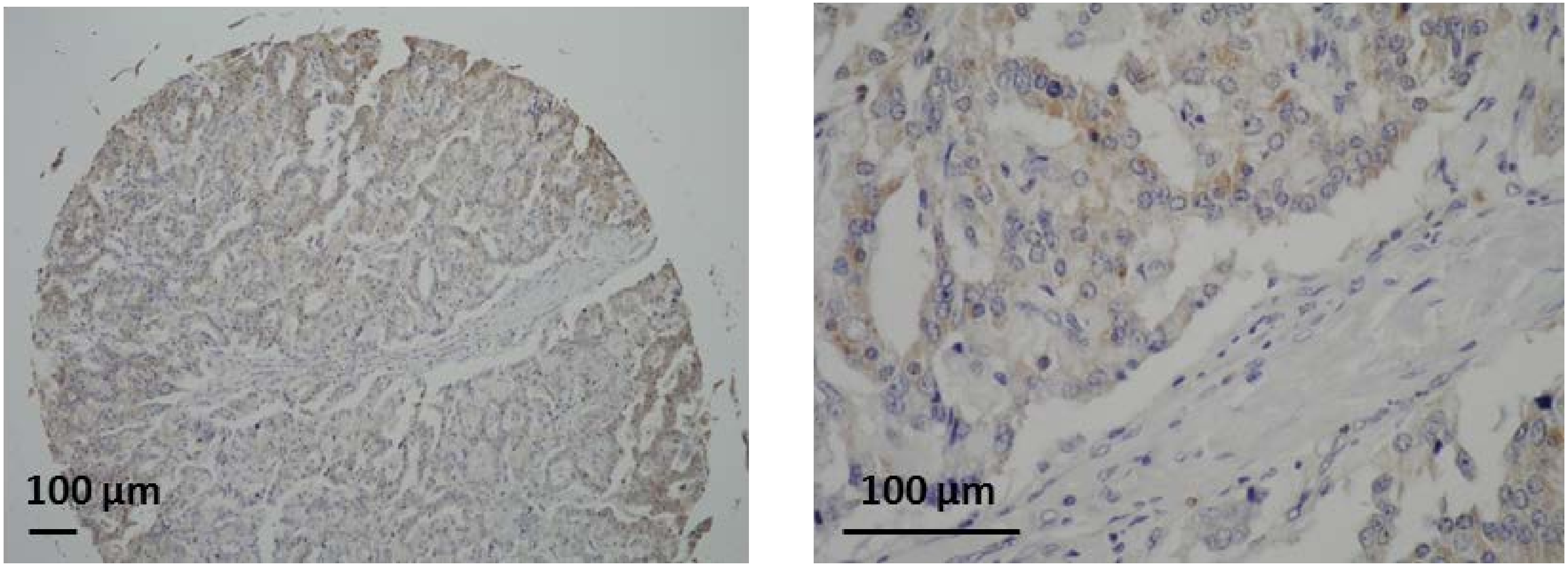

Figure 2S: RNLS expression in neuroendocrine tumor of the pancreas. Representative immunohistochemistry staining for RNLS protein expression (brown) using m28-RNLS in a human neuroendocrine tumor.

3S

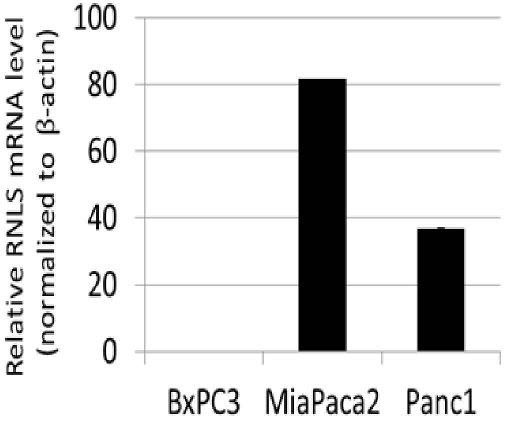

4S

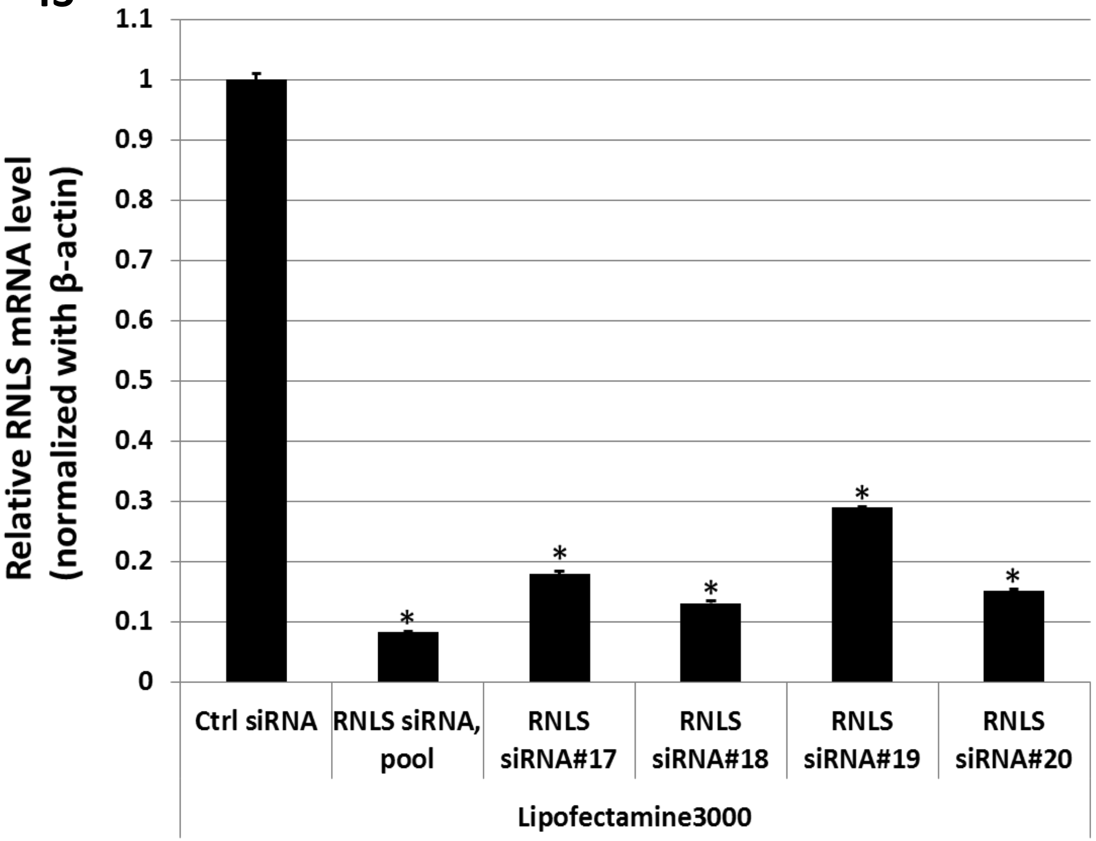

5S

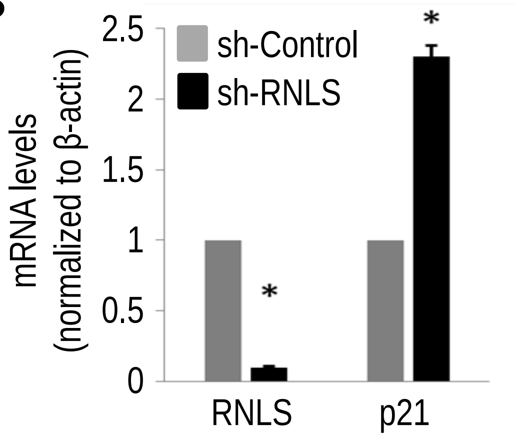

Figure 3S: Renalase mRNA level in pancreatic cancer cell lines. RNLS mRNA level measured by RT-qPCR.

Figure 4S: Knockdown of RNLS expression using siRNA. MiaPaCa2 cells were transfected with indicated siRNA for 3days and RNLS mRNA levels were measured by RTqPCR.

Figure 5S: Knockdown of RNLS expression using shRNA. Panc1 cells were stably transfected with shRNA construct to specifically knockdown RNLS. The RNLS and p21 mRNA levels were measured by RT-qPCR.

Supplement 6S

Figure 6S: Inhibition of RNLS by monoclonal antibody (clone 28-4) induces PDAC cell apoptosis. Panc1 cells were treated with 28-4 for days followed by flow cytometry analysis. Numbers in the upper left quadrants of dot plots indicate the percentage of necrotic cells; numbers in the upper right quadrants, late apoptotic cells; numbers in the lower left quadrants, viable cells; numbers in the lower right quadrants, early apoptotic cells.

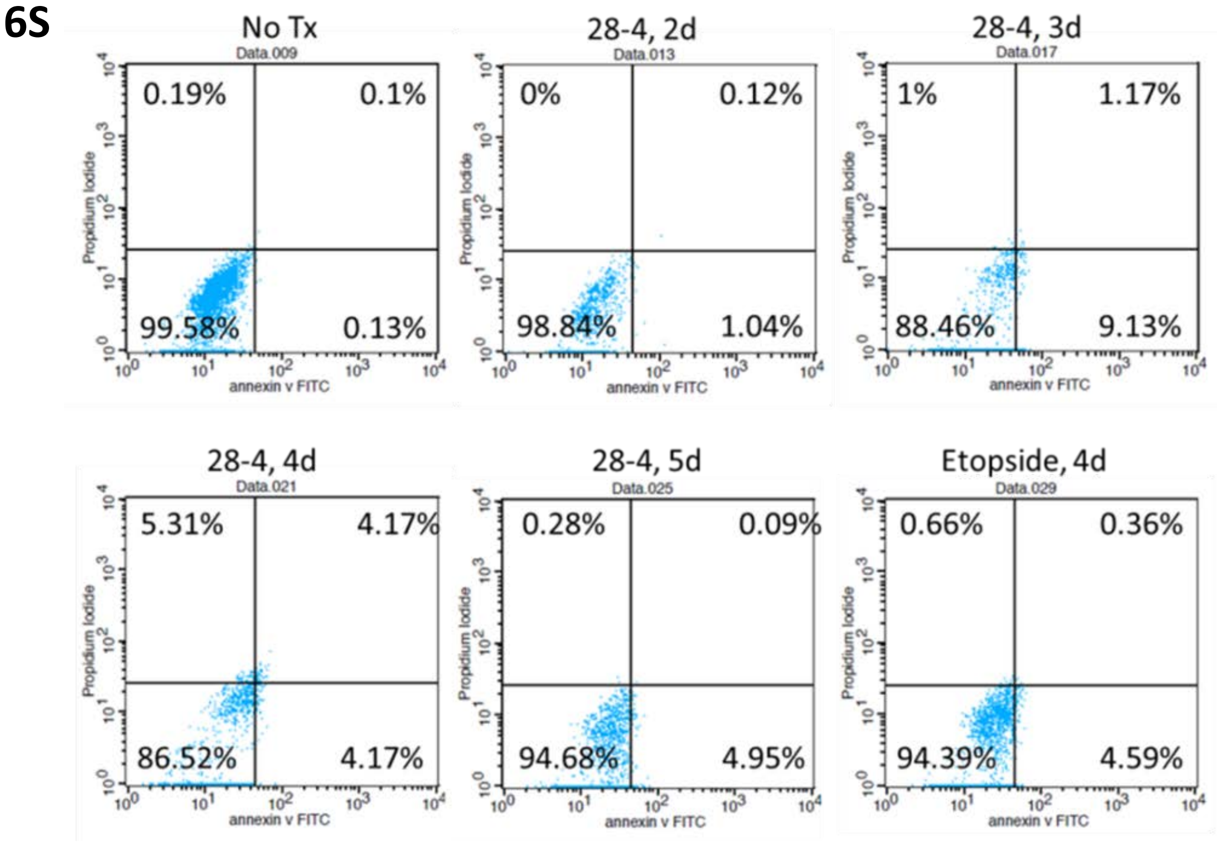

Supplement 7S

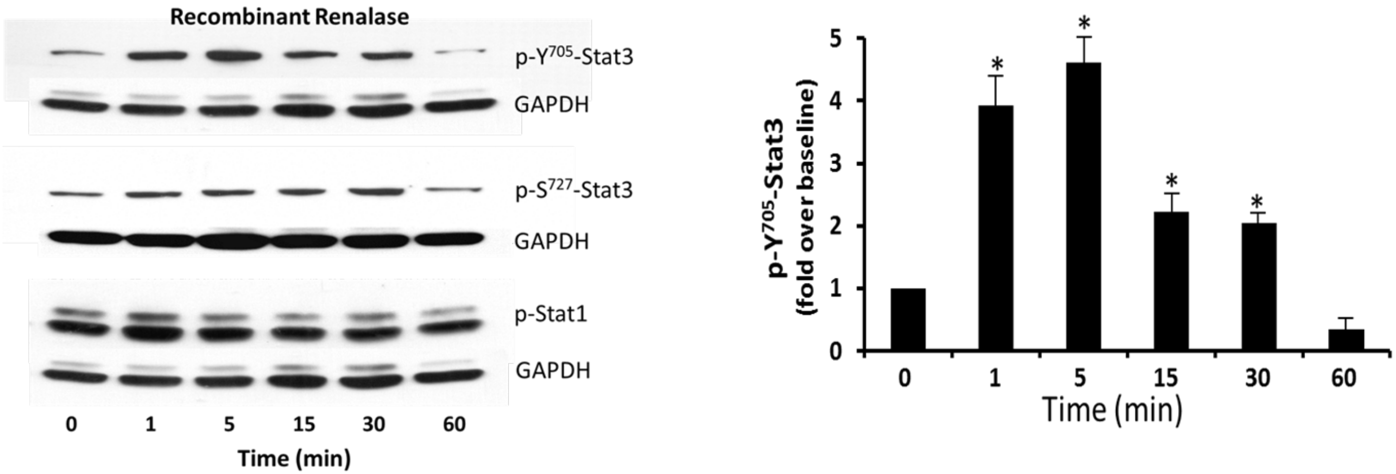

Figure 7S: RNLS activates STAT3 in human embryonic kidney cells (HK-2). *Left panel*: Cells were treated recombinant (30 mcg/ml) and STAT3 and STAT1 phosphorylation determined by Western blotting. *Right panel*: quantification of STAT3 phosphorylation at tyrosine 705 with normalization to GADPH. n-=5, * indicates $p<0.01$.

Supplement 8S, 9S

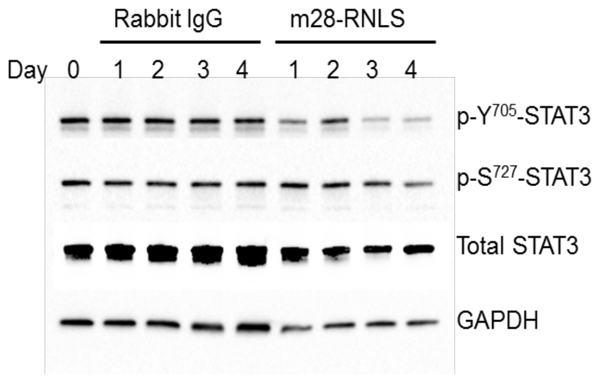

8S: m28-RNLS inhibits STAT3 phosphorylation; Panc1 cells in culture treated with either rabbit IgG or anti- RNLS monoclonal m28-RNLS for up to 4 days, and STAT3 phosphorylation assessed by western blot; p-Ser^{727}-STAT3: phosphorylation at serine 727, p-Y^{705}-STAT3: phosphorylation at tyrosine 705; GAPDH loading control; representative study.

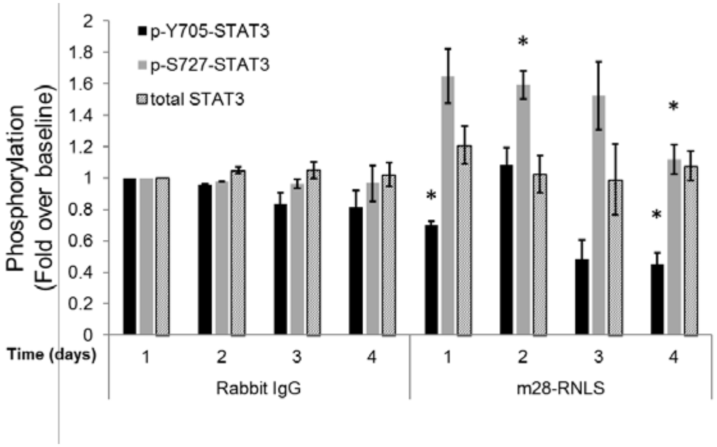

9S: Quantification of STAT3 phosphorylation with m28-RNLS; signals normalized to GAPDH loading control; n=3, *=P<0.05. m28-RNLS decreases STAT3 phosphorylation at tyrosine 705 by ~60%.

Supplement Table 1S

Table 1S.: Characteristics of patient cohort with PDAC

Characteristics		Number /Total number
Gender		
	Female	24/69 (34.8%)
	Male	45/69 (65.2%)
Age (years)		
	Median	61 (36-85)
	36-50	14/69 (20.3%)
	51-69	39/69 (56.5%)
	70-85	16/69 (23.2%)
Tumor Grade		
	1	1/69 (1.4%)
	2	48/69 (69.6%)
	3	15/69 (21.7%)
	4	1/69 (1.4%)
	Unknown	4/69 (5.8%)
Survival (Months)		
	0-12	29/69 (42%)
	13-24	9/69 (13%)
	25-48	18/69 (26%)
	49-87	13/69 (19%)
